# Deep transcranial magnetic stimulation for the treatment of auditory hallucinations: a preliminary open-label study

**DOI:** 10.1186/1744-859X-10-3

**Published:** 2011-02-09

**Authors:** Oded Rosenberg, Yiftach Roth, Moshe Kotler, Abraham Zangen, Pinhas Dannon

**Affiliations:** 1 Beer Ya'akov Mental Health Center affiliated to Sackler School of Medicine, University of Tel Aviv, Tel Aviv, Israel; 2 The Weizmann Institute of Science, Rehovot, Israel

## Abstract

**Background:**

Schizophrenia is a chronic and disabling disease that presents with delusions and hallucinations. Auditory hallucinations are usually expressed as voices speaking to or about the patient. Previous studies have examined the effect of repetitive transcranial magnetic stimulation (TMS) over the temporoparietal cortex on auditory hallucinations in schizophrenic patients. Our aim was to explore the potential effect of deep TMS, using the H coil over the same brain region on auditory hallucinations.

**Patients and methods:**

Eight schizophrenic patients with refractory auditory hallucinations were recruited, mainly from Beer Ya'akov Mental Health Institution (Tel Aviv university, Israel) ambulatory clinics, as well as from other hospitals outpatient populations. Low-frequency deep TMS was applied for 10 min (600 pulses per session) to the left temporoparietal cortex for either 10 or 20 sessions. Deep TMS was applied using Brainsway's H1 coil apparatus. Patients were evaluated using the Auditory Hallucinations Rating Scale (AHRS) as well as the Scale for the Assessment of Positive Symptoms scores (SAPS), Clinical Global Impressions (CGI) scale, and the Scale for Assessment of Negative Symptoms (SANS).

**Results:**

This preliminary study demonstrated a significant improvement in AHRS score (an average reduction of 31.7% ± 32.2%) and to a lesser extent improvement in SAPS results (an average reduction of 16.5% ± 20.3%).

**Conclusions:**

In this study, we have demonstrated the potential of deep TMS treatment over the temporoparietal cortex as an add-on treatment for chronic auditory hallucinations in schizophrenic patients. Larger samples in a double-blind sham-controlled design are now being preformed to evaluate the effectiveness of deep TMS treatment for auditory hallucinations.

**Trial registration:**

This trial is registered with clinicaltrials.gov (identifier: NCT00564096).

## Introduction

Schizophrenia is usually accompanied by reality distortion followed by frequent delusions and hallucinations. Hallucinations may be both visual and auditory, while the latter is more frequent. Auditory hallucinations are usually expressed by voices speaking to or about the patient [1]. The biochemical mechanisms behind auditory hallucinations (AHs) remain elusive. Generally, AHs may be considered to stem from a default monitoring of inner states. As a result, the individual mislabels the inner speech as non-self [2].

Auditory hallucinations are reported by 50% to 70% of patients with schizophrenia, and the majority of cases are successfully treated with antipsychotic medications. However, 25% to 30% of hallucinating schizophrenic patients are refractory to antipsychotic medications, and therefore patients suffer associated distress, functional disability, lack of behavioral control [3] and violent behavior [4]. It has also been known to be a contributing factor in up to 25% of cases of serious suicide attempts [5].

Transcranial magnetic stimulation (TMS) is a non-invasive tool that stimulates nerve cells in superficial areas of the brain. TMS, which was first introduced in 1985 [6], induces a magnetic field that can produce a substantive electrical field in the brain causing depolarization of nerve cells, which results in the stimulation or disruption of local brain activity. TMS may be applied as a single stimulus, or repeated many times per seconds (rTMS), with variation in intensity, site and orientation of the magnetic field [7]. The first report of rTMS treatment for auditory hallucinations was described in 1999 by Hoffman *et al. *[8]. In that study, rTMS was applied over the left temporoparietal cortex of three patients over 4 days (for 4, 8, 12 and 16 min). Hoffman *et al. *reported an improvement in auditory hallucination severity in those patients, as rated on a visual analogue scale (VAS) [8]. Since then, several studies have used rTMS to treat auditory hallucinations in schizophrenic patients, targeting almost exclusively the left temporoparietal cortex, with mixed results [3,4,7,9,10]. The physiological basis of the rTMS-induced beneficial effect on auditory hallucinations is not well understood, but may reflect reduced pyramidal neuron excitability or neuroplasticity changes analogous to those associated with long-term depression [3,4,10]. Imaging studies of patients with of auditory hallucinations demonstrated increased blood flow in the speech perception areas of the brain, such as the superior temporal cortex of the dominant hemisphere and the superior temporal cortex bilaterally [11], and therefore, neuronal hyperactivity in these areas has been associated with AHs. Overactivation of the left temporoparietal cortex, which is critical to speech perception and is easily accessible to rTMS, has been implicated to be involved in the onset of auditory hallucinations [10]. In a 2003 study, Hoffman *et al. *detected improvement primarily in frequency and attentional salience of hallucinations, which were also associated with modest overall clinical improvement, but with no negative effects of rTMS on cognition [4].

The H1 coil, used for deep TMS, has been shown to be effective in the treatment of major depression [12-14]. Deep TMS coils are designed to maximize the electrical field in deep brain tissues by the summation of separate fields projected into the skull from several points around its periphery [15]. The device is planned to minimize the accumulation of electrical charge on the surface of the brain, which can give rise to an electrostatic field that might reduce the magnitude of the induced electric field both at the surface and inside, and reduce the depth penetration of the induced electric field [16]. Deep TMS could be more effective than rTMS due to the larger and deeper spread of field it can produce [15]. In our study we examined the efficacy of deep TMS over the left temporoparietal cortex for the treatment of auditory hallucinations in refractory schizophrenic patients.

## Methods

### Participants

Eight participants (an equal number of males and females) were recruited to this study via outpatient clinics all over Israel. All patients gave written informed consent to take part in the study, which was approved by the Beer-Ya'akov Mental Health Center Ethics Committee and the Israeli Ministry of Health. Inclusion criteria were: age between 18 to 65, ability to sign an informed consent, meeting Diagnostic and Statistical Manual of Mental Disorders, fourth edition text revision (DSM-IV-TR) criteria for schizophrenia/schizoaffective disorder, experiencing auditory hallucinations at least five times per day, and use of a stable antipsychotic medication for at least 1 month prior to enrollment.

Participant ages ranged between 28 to 62 years (average 28.8 years). Six patients were diagnosed with schizophrenia and two were diagnosed with schizoaffective disorder. Seven were outpatients and one an inpatient. Hallucinations had persisted for an average of 11 years, despite adequate trials with an average of 4.75 (SD ± 1.9) antipsychotic medications prior to study entry. The auditory hallucinations of six patients were also resistant to treatment with an average dose of 470 mg/day clozapine (SD ± 75.8 mg). All participants were on antipsychotic medication during the study, with their dosage of medication being kept stable throughout the study. Demographic data for all patients is presented in Table 1.

**Table 1:** Demographic data

**Patient no**.	Sex	Age	Status	Education, years	Diagnosis	Age of disease onset	Number of past hospitalizations	Time elapsed since present episode of auditory hallucinations started, years	No. of antipsychotic medications to which auditory hallucinations were resistant
1	M	30	Outpatient	11	Schizophrenia	19	4	11	6

2	F	62	Inpatient	13	Schizoaffective	53	3	9	6

3	M	58	Outpatient	10	Schizophrenia	18	>10	29	2

4	F	47	Outpatient	12	Schizoaffective	25	>10	5	5

5	M	28	Day care	12	Schizophrenia	27	2	1	6

6	M	37	Outpatient	13	Schizophrenia	20	7	18	7

7	F	54	Outpatient	10	Schizophrenia	42	7	12	4

8	F	55	Outpatient	9	Schizophrenia	27	2	5	2

Exclusion criteria for deep TMS are essentially the same as those for rTMS, including: neurosurgery, brain trauma, patients suffering from chronic medical conditions of any sort, history of current hypertension, history of seizure or heart convulsion, history of epilepsy or seizure in first degree relatives, history of head injury, history of any metal objects in the head area (other than the mouth), known history of any metallic particles in the eye, implanted cardiac pacemaker or any intracardiac lines, implanted neurostimulators, surgical clips or any medical pumps, history of frequent or severe headaches, history of migraine, history of hearing loss, known history of cochlear implants, history of drug abuse or alcoholism, pregnancy (tested by β-human chorionic gonadotropin test) or not using a reliable method of birth control, systemic and metabolic disorders, inadequate communication skills or being under custodial care.

### Deep TMS procedure

We performed the treatments with Brainsway's H1 coil (Brainsway, Jerusalem, Israel), which was checked in a safety study with healthy volunteers [17], and in a clinical study for the treatment of major depression (Levkovitz *et al. *[14]). The H1 coil detailed configuration and electric field distribution maps are described in Roth *et al. *[17]. Deep TMS was administered by a Brainsway's H1 coil, connected to a Magstim Rapid^2 ^stimulator (Magstim, Whitland, UK). The resting motor threshold for each participant was obtained by stimulation to the left motor cortex, and defined as the minimum stimulator output intensity that causes a motor response (that is, twitching of the contralateral abductor policis brevis (APB) muscle in the hand).

**Figure 1:**
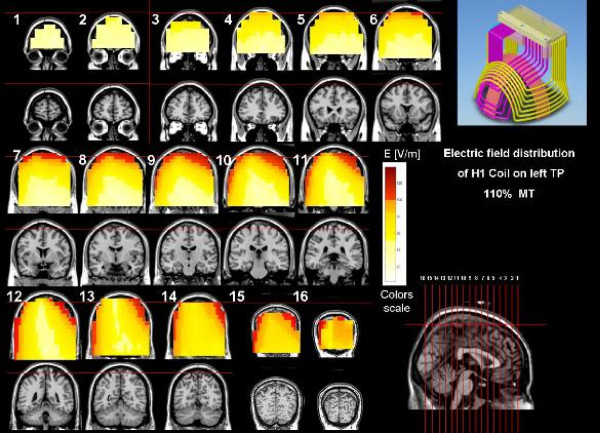
**Electric field distribution maps of the H1 coil when placed during stimulation over the left temporoparietal cortex, at an intensity of 110% of a typical abductor policis brevis (APB) motor threshold**. The images are based on electric field measurements in a phantom head model filled with saline water at physiological concentration.

The coil was then moved 4.5 cm posteriorly and 6.5 cm laterally towards the left shoulder of the patient. In this position, the maximal electric field produced by the coil is at the left temporoparietal cortex (Figure 1).

Patients were treated with 10 min of deep H coil TMS to the left temporoparietal cortex at a frequency of 1 Hz with 110% motor threshold for either 10 or 20 working days (Five days a week and two days weekend interval) (Table 2).

**Table 2:** Treatment parameters

**Patient no**.	Motor threshold	Pulses per session	No. of sessions
1	110%	600	10

2	110%	600	10

3	110%	600	10

4	110%	600	10

5	110%	600	10

6	110%	600	20

7	110%	600	20

8	110%	600	20

### Patient assessment

Diagnoses were made by trained psychiatrists using a semistructured clinical interview based on DSM-IV-TR criteria [Structured Clinical Interview for DSM-IV Axis I Disorders, version 2 (SCID-II)], during which patients main demographic and clinical characteristics were collected. Each patient was evaluated within 24 h prior to TMS study session, and post treatment within 24 h of the last session, using the Auditory Hallucinations Rating Scale (AHRS) developed by Hoffman *et al. *[4], the Scale for the Assessment of Positive Symptoms scores (SAPS; [10]), the Clinical Global Impressions (CGI) scale, and the Scale for the Assessment of Negative Symptoms (SANS).

In addition, patients were evaluated with AHRS and all other rating scales within 1 day after the last treatment session, and at 1 week and 1 month follow-up sessions.

## Results

**Figure 2:**
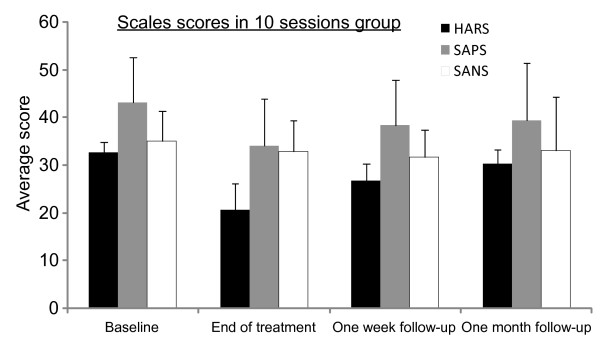
**Average scores of Auditory Hallucinations Rating Scale (AHRS), Scale for the Assessment of Positive Symptoms (SAPS) and Scale for Assessment of Negative Symptoms (SANS) (with standard error of mean (SEM)) 1 day before treatment (baseline), 1 day after last session (end of treatment), 1 week after last session and at 1 month after last session in the 10-session group**.

A total of 5 patients were first treated for 10 days with deep H coil TMS over the left temporoparietal cortex at a frequency of 1 Hz for 10 min using an intensity of 110% of the motor threshold. For those patients, average AHRS at the end of treatment improved by 34.5% (SD ± 38.2%) compare to baseline, including one patient for whom auditory hallucination ceased completely for 2 days. Average SAPS improved by 23.1% (SD ± 18. 9%), and there was also minor reduction of 11.2% (SD ± 10.4%) in CGI score and 9.2% reduction (SD ± 10%) in SANS score. However, during follow-up all results gradually returned to baseline levels and the effect of hallucination amelioration was lost almost completely (Figure 2). Therefore, the number of sessions was increased for the next 3 patients to 20 sessions.

**Figure 3:**
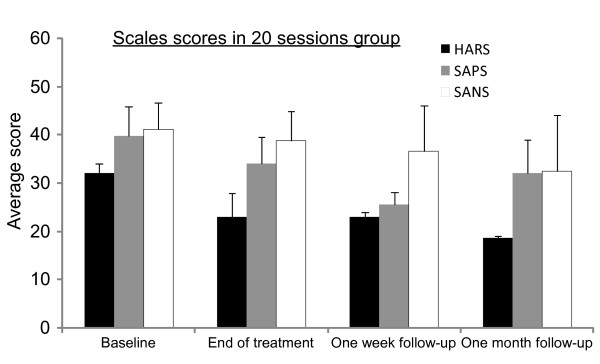
**Average scores of Auditory Hallucinations Rating Scale (AHRS), Scale for the Assessment of Positive Symptoms (SAPS) and Scale for Assessment of Negative Symptoms (SANS) (with standard error of mean (SEM)) 1 day before treatment (baseline), 1 day after last session (end of treatment), 1 week after last session and at 1 month after last session in the 20-session group**.

In these patients (patients 6-8), average AHRS was improved at the end of treatment by 27.8% (SD ± 26.2%), average SAPS score improved by 13.75% (SD ± 12.3%), and there was also a minor reduction of 6.5% (SD ± 7.3) in SANS score. One patient did not improve and was lost to follow-up after treatment. However, in contrast to the first five patients, in the remaining two patients symptom scores kept improving such that at the 1 month follow-up the average change in AHRS and SAPS scores reached a reduction of 42.6% and 17.9%, respectively (Figure 3). Auditory hallucination scale paired *t *test values for all eight patients at baseline were as follows: *P *= 0.039 at 24 h follow-up, *P *= 0.004 at 1 week follow-up and *P *= 0.029 at 1 month follow-up (Table 3).

**Table 3:** Evaluation results

**Patient no**.	Baseline scores				End of treatment scores (follow-up 1)					Scores 1 week from last session (follow-up 2)					Scores 1 month from last session (follow-up 3)				
	
	CGI-S	CGI-I	CGI-I	CGI-S	SANS	SAPS	AHRS	CGI-S	SANS	SAPS	AHRS	SANS	SAPS	AHRS	CGI-I	CGI-S	SANS	SAPS	AHRS
1	5	3	3	5	32	11	26	5	39	22	22	41	31	33	3	5	33	22	28

2	5	3	3	4	25	45	23	4	25	49	24	27	49	27					

3	6	3	4	5	46	69	31	5	48	65	32	50	76	37	4	5	57	68	33

4	5	2	2	5	41	39	37	4	41	19	0	42	39	38			39	50	37

5	4	4	2	3	14	27	16	4	11	15	25	15	20	28			3	17	23

6	4	3	4	4	46	23	22	4	48	23	14	48	29	29	5	4	44	25	18

7	5	5						5	41	40	31	45	40	31					

8	5	3	4	5	27	28	24	5	27	39	24	30	50	36	3	5	21	39	19

**Average**	**4.87**	**3.25**	**3.14**	**4.42**	**33**	**34.57**	**25.57**	**4.5**	**35**	**34**	**21.5**	**37.25**	**41.75**	**32.37**	**3.75**	**4.75**	**32.83**	**36.83**	**26.33**

**SD**	**0.64**	**0.88**	**0.89**	**0.78**	**12**	**18.72**	**6.75**	**0.53**	**12.9**	**17.31**	**10.3**	**12.11**	**17.13**	**4.27**	**0.95**	**0.5**	**18.85**	**19.5**	**7.68**

AHRS = Auditory Hallucinations Rating Scale; CGI(-I/-S) = Clinical Global Impression (Improvement/Severity); SANS = Scale for Assessment of Negative Symptoms; SAPS = Scale for the Assessment of Positive Symptoms; SD = standard deviation.

### Side effects

Treatment was very well tolerated. One patient experienced headache after one session, which subsided after administration of 500 mg of paracetamol.

## Discussion

All patients but one improved with deep TMS, and one patient's auditory hallucinations ceased completely. The results at the end of treatment were better in the group receiving 10 sessions; however, this group's symptom scores gradually returned to baseline levels during follow-up. Conversely, in 2 out of 3 patients receiving 20 sessions, we observed less improvement at the end of treatment but a further improvement during follow-up, reaching a considerable reduction of auditory hallucinations at the 1 month follow-up. Considering the resistance of auditory hallucinations to treatment in these patients (failure of 4.75 trials of antipsychotic medications on average), this study may mark a direction for future explorations using deep TMS, in which sham-controlled studies would be crucial to demonstrate efficacy.

An electroconvulsive therapy study of 253 patients with schizophrenia found greater severity of baseline negative symptoms to be predictive of poor outcome [18]. In our study we observed no correlation between baseline negative symptoms as judged by SANS and amelioration of auditory hallucinations. Loo *et al. *[19] noted that examinations of individual-controlled trials reveal that a substantial proportion of rTMS studies for the treatment of auditory hallucinations did not find rTMS superior to sham stimulations. The authors also noted that although most trials have involved the administration of rTMS to the left temporoparietal cortex, it is far from conclusive that abnormalities associated with auditory hallucinations are specific to the left hemisphere [19]. There is some evidence that the pathology of auditory hallucinations involves not only the left hemisphere, but also the right one [11]. According to Vercammen *et al*., evidence from neuroimaging studies suggests a potential for bilateral temporal cortex involvement in the genesis of auditory hallucinations. Left superior temporal areas are hypothesized to be involved in speech perception during the hallucinations, and the right temporal cortex may be more associated with the processing of prosody and emotional salience, which is often expressed in the derogatory and hostile content of the hallucinations [20]. Schreiber *et al*., in a case study, showed that daily right prefrontal rTMS for 20 days at 10 Hz frequency with 90% motor threshold may induce a general clinical improvement in the brain function of patients with schizophrenia [21]. The advantage of left-sided or right-sided stimulation might be individually determined, depending on the individual underlying pathophysiology. rTMS shows the best results when guided by functional MRI to areas of activation during hallucinations, whether in the left or right hemisphere [22].

## Limitations

The limitations of our study are the small number of patients, lack of a sham control group, the rater not being blind and the heterogeneity of treatment (5 patients underwent 10 sessions while 3 underwent 20 sessions).

## Conclusions

Our preliminary results showed a significant improvement in our patient group. The small number of patients in our study precludes a conclusion regarding deep TMS efficacy, even though it marks a direction for possible future studies. We believe that a future large-scale, double-blind, sham-controlled study, targeting various brain regions, could clarify the effectiveness of deep TMS in the treatment of resistant auditory hallucinations.

## Competing interests

PD and OR received an unrestricted educational grant for TMS research from Brainsway. AZ serves as a research consultant and has financial interest in Brainsway. MK declares no competing interests. YR is working as a research consultant at Brainsway and has a financial interest in Brainsway.

## Authors' contributions

RO participated in the deep TMS treatments described in the text, participated in writing the basic draft of the paper and rewriting the text according to coauthor suggestions, participated in drafting the discussion and conclusions, and participated in clinical evaluations. KM participated in final approval of the manuscript. ZA participated by making extensive suggestions, advised on background, methods, discussion and conclusions, and guided the paper scientifically. DP participated by making contributing remarks and suggestions on how to revise the text, including the discussion and conclusions, closely supervised the deep TMS sessions as well as conducted part of the deep TMS treatments. YR designed the H1 coil, created electric field distribution maps of the H1 coil, contributed remarks and suggestions to revising the text, including the discussion and conclusions. All authors read and approved the final manuscript. RO works at the Beer Ya'akov Mental Health Center and is paid by the research fund of the Beer Ya'akov Mental Health Center. KM serves as the director of the Beer Ya'akov Mental Health Center. ZA works at the Department of Neurobiology of the Weizmann Institute of Science and also serves as a research consultant for Brainsway. DP is head of the research department of Beer Ya'akov Mental Health Center and head of the electroconvulsive therapy unit of the Beer Ya'akov Mental Health Center. PD is paid by by Beer Ya'akov Mental Health Center. YR works as a research consultant for Brainsway.

## References

[B1] Hugdahl K Løberg EM Jørgensen HA Lundervold A Lund A Green MF Rund B Left hemisphere lateralization of auditory hallucinations in schizophrenia: a dichotic listening study Cogn Neuropsychiatry 2008 13 166 179 10.1080/13546800801906808 18302028

[B2] Tranulis C Sepehry AA Galinowski A Stip E Should we treat auditory hallucinations with repetitive transcranial magnetic stimulation? A metaanalysis Can J Psychiatry 2008 53 577 586 18801220 10.1177/070674370805300904

[B3] Poulet E Brunelin J Bediou B Bation R Forgeard L Dalery J d'Amato T Saoud M Slow transcranial magnetic stimulation can rapidly reduce resistant auditory hallucinations in schizophrenia Biol Psychiatry 2005 57 188 191 10.1016/j.biopsych.2004.10.007 15652879

[B4] Hoffman RE Hawkins KA Gueorguieva R Boutros NN Rachid F Carroll K Krystal JH Transcranial magnetic stimulation of left temporoparietal cortex and medication-resistant auditory hallucinations Arch Gen Psychiatry 2003 60 49 56 10.1001/archpsyc.60.1.49 12511172

[B5] Shergill SS Murray RM McGuire PK Auditory hallucinations: a review of psychological treatments Schizophr Res 1998 32 137 150 10.1016/S0920-9964(98)00052-8 9720119

[B6] Barker AT Jalinous R Freeston IL Non-invasive magnetic stimulation of human motor cortex Lancet 1985 8437 1106 1107 10.1016/S0140-6736(85)92413-4 2860322

[B7] Fitzgerald PB Daskalakis ZJ A review of repetitive transcranial magnetic stimulation use in the treatment of schizophrenia Can J Psychiatry 2008 53 567 576 18801219 10.1177/070674370805300903

[B8] Hoffman RE Boutros NN Berman RM Roessler E Belger A Krystal JH Charney DS Transcranial magnetic stimulation of left temporoparietal cortex in three patients reporting hallucinated "voices" Biol Psychiatry 1999 46 130 132 10.1016/S0006-3223(98)00358-8 10394483

[B9] Thirthalli J Bharadwaj B Kulkarni S Gangadhar BN Kharawala S Andrade C Successful use of maintenance rTMS for 8 months in a patient with antipsychotic-refractory auditory hallucinations Schizophr Res 2008 100 351 352 10.1016/j.schres.2008.01.003 18252271

[B10] Poulet E Brunelin J Kallel L Bediou B Dalery J D'amato T Saoud M Is rTMS efficient as a maintenance treatment for auditory verbal hallucinations? A case report Schizophr Res 2006 84 183 184 10.1016/j.schres.2006.02.014 16580181

[B11] Lee SH Kim W Chung YC Jung KH Bahk WM Jun TY Kim KS George MS Chae JH A double blind study showing those two weeks of daily repetitive TMS over the left or right temporoparietal cortex reduces symptoms in patients with schizophrenia who are having treatment-refractory auditory hallucinations Neurosci Lett 2005 376 177 181 10.1016/j.neulet.2004.11.048 15721217

[B12] Rosenberg O Shoenfeld N Zangen A Kotler M Dannon PN Deep TMS in a resistant major depressive disorder: a brief report Depress Anxiety 2010 27 465 469 10.1002/da.20689 20455247

[B13] Rosenberg O Zangen A Stryjer R Kotler M Dannon PN Response to Deep TMS in depressive patients with previous electroconvulsive treatment Brain Stimul 2010 3 211 217 10.1016/j.brs.2009.12.001 20965450

[B14] Levkovitz Y Harel EV Roth Y Braw Y Most D Katz LN Sheer A Gersner R Zangen A Deep TMS over prefrontal cortex: evaluation of antidepressant and cognitive effects in depressive patients Brain Stimul 2009 2 188 200 10.1016/j.brs.2009.08.002 20633419

[B15] Zangen A Roth Y Voller B Hallett M Transcranial magnetic stimulation of deep brain regions: evidence for efficacy of the H-coil Clin Neurophysiol 2005 116 775 779 10.1016/j.clinph.2004.11.008 15792886

[B16] Roth Y Zangen A Hallett M A coil design for transcranial magnetic stimulation of deep brain regions J. Clin Neurophysiol 2002 19 361 370 10.1097/00004691-200208000-00008 12436090

[B17] Levkovitz Y Roth Y Harel EV Braw Y Sheer A Zangen A A randomized controlled feasibility and safety study of deep transcranial magnetic stimulation Clin Neurophysiol 2007 118 2730 2744 10.1016/j.clinph.2007.09.061 17977787

[B18] Chanpattana W Sackeim HA Electroconvulsive therapy in treatment-resistant schizophrenia: prediction of response and the nature of symptomatic improvement J ECT 2010 26 289 298 10.1097/YCT.0b013e3181cb5e0f 20375701

[B19] Loo CK Sainsbury K Mitchell P Hadzi-Pavlovic D Sachdev PS A sham-controlled trial of left and right temporal rTMS for the treatment of auditory hallucinations Psychol Med 2009 6 1 6 10.1017/S0033291709990900 19656432

[B20] Vercammen A Knegtering H Bruggeman R Westenbroek HM Jenner JA Slooff CJ Wunderink L Aleman A Effects of bilateral repetitive transcranial magnetic stimulation on treatment resistant auditory-verbal hallucinations in schizophrenia: a randomized controlled trial Schizophr Res 2009 14 172 179 10.1016/j.schres.2009.07.013 19679450

[B21] Schreiber S Dannon PN Goshen E Amiaz R Zwas TS Grunhaus L Right prefrontal rTMS treatment for refractory auditory command hallucinations - a neuroSPECT assisted case study Psychiatry Res 2002 116 113 117 10.1016/S0925-4927(02)00065-3 12426038

[B22] Sommer IE de Weijer AD Daalman K Neggers SF Somers M Kahn RS Slotema CW Blom JD Hoek HW Aleman A Can fMRI-guidance improve the efficacy of rTMS treatment for auditory verbal hallucinations? Schizophr Res 2007 93 406 408 10.1016/j.schres.2007.03.020 17478084

